# Leveraging wearables to safeguard workers in a hotter climate

**DOI:** 10.3389/fpubh.2026.1867796

**Published:** 2026-09-01

**Authors:** Margaret C. Morrissey, Jason Kai Wei Lee, Ian Rollo, Yannis Pitsiladis

**Affiliations:** 1 Department of Health Sciences, Providence College, Providence, RI, United States; 2 Korey Stringer Institute, University of Connecticut, Storrs, CT, United States; 3 National University of Singapore Yong Loo Lin School of Medicine, Singapore, Singapore; 4 Heat Resilience & Performance Centre, Yong Loo Lin School of Medicine, National University of Singapore, Singapore, Singapore; 5 Human Potential Translational Research Program, Yong Loo Lin School of Medicine, National University of Singapore, Singapore, Singapore; 6 Department of Physiology, Yong Loo Lin School of Medicine, National University of Singapore, Singapore, Singapore; 7 Gatorade Sports Science Institute, Barrington, IL, United States; 8 School of Sport, Exercise and Health Sciences, Loughborough University, Loughborough, United Kingdom; 9 Centre for Exercise Science and Medicine (CESAME), Hong Kong Baptist University, Kowloon Tsai, Hong Kong SAR, China

**Keywords:** athlete safety, climate change, heat stress, occupational heat exposure, physiological monitoring

## Abstract

Rises in global temperatures have threatened the health, safety, and productivity of workers who perform physically demanding work. Traditionally, occupational heat illness prevention plan have relied on environmental monitoring and administrative controls, however, these approaches do not account for the considerable intra- and inter-variability in physiological responses to extreme heat. In contrast, elite sport has served as a valuable test bed for investigating thermoregulation, exertional heat illness, and the feasibility of real-time physiological monitoring. Experiences from recent Olympic Games have demonstrated that integrated environmental and physiological monitoring systems can operate at scale under extreme heat conditions, while also highlighting important technological, operational, and governance limitations. Translating these advances to occupational settings presents both opportunities and challenges because workers differ substantially from athletes in age, health status, heat acclimatisation, work demands, exposure duration, and access to medical oversight. Furthermore, implementation is complicated by concerns regarding data governance, privacy, informed consent, device validity, interpretation of physiological information, and the use of physiological thresholds to guide workplace decisions. This commentary examines lessons learned from elite sport and discusses their application to occupational heatsafety programs. We propose a tiered adoption framework that aligns physiological monitoring complexity with organizational capacity, technical expertise, and governance requirements, ranging from foundational heat-safety practices to advanced multimodal physiological monitoring under professional oversight. Physiological monitoring should complement, but not replace, established heat-stress prevention strategies and be implemented within clearly defined operational and ethical boundaries. Future research should focus on validating wearable technologies in occupational environments, developing individualized decision frameworks, and establishing governance structures that support responsible implementation.

## Introduction

Extreme heat is an increasingly significant threat to health, performance, and productivity in both sport and occupational environments (1, 2). Rising global temperatures have intensified physiological strain during exercise and work, with 2024 confirmed as the warmest year on record at 1.28 °C above the 1951–1980 baseline (3). Projections from the United Nations (UN) Environment Programme indicate that current emission trajectories could produce as much as 2.8 °C of warming by 2100, surpassing thresholds associated with severe global health and environmental impacts (4). Heatwaves already disrupt communities and economies, and the UN has called for coordinated global action to mitigate associated risks. The UN has identified workers in physically demanding and outdoor occupations as a particularly vulnerable population to the health impacts of extreme heat (5).

Despite decades of occupational heat guidance, most existing frameworks remain focused primarily on environmental conditions, relying on indices and administrative controls that do not capture individual differences in physiological vulnerability. Workers differ substantially in health status, age, physical fitness, and heat acclimatization, all of which can markedly influence physiological responses to heat exposure. Furthermore, many workers are exposed to prolonged or uncompensable heat conditions, while numerous occupations require heavy protective clothing or sustained effort under constrained conditions (1, 6–8). Despite workers’ heightened vulnerability to excessive heat strain, there is limited understanding of which tasks and worker populations are at greatest risk, and evidence-based heat-mitigation strategies remain inadequately characterized across industries (9). This lack of field-based evidence is partly attributable to the practical challenges associated with implementing physiological monitoring within existing occupational heat-stress management practices.

In contrast, sports science has developed extensive insight into thermoregulation and exertional heat illness (EHI) through controlled experimentation and decades of applied field research (10–13). Because elite sport combines high metabolic heat production with structured medical oversight and standardized monitoring protocols, it provides a unique model for evaluating physiology-based heat safety strategies. These contrasting contexts create opportunities for knowledge transfer between sport and occupational health domains, provided that translation remains realistic and governed appropriately. In other words, sport has demonstrated feasibility and governance models for physiological monitoring in heat, but translation to occupational settings requires structured operational frameworks and strict ethical guidelines. Therefore, the purpose of this commentary was to propose an operational framework for implementing physiological monitoring systems in the workplace. We highlight the evidence and feasibility of physiology-based monitoring in sport and discuss its translation to occupational settings as a strategy to improve individualized heat-risk management and promote wider adoption of this practice.

## What sport learned: evidence, limits, and feasibility of physiology-based monitoring

Evidence generated in elite sport provides a detailed empirical foundation for understanding how physiological monitoring can inform heat-safety decisions under extreme environmental stress. Elite sport represents a uniquely controlled yet physiologically extreme environment in which athletes generate high metabolic heat loads under intense environmental stress while operating within structured medical oversight. These characteristics have enabled systematic evaluation of real-time monitoring systems in ways that are difficult in occupational settings.

The Tokyo 2020 Olympic Games illustrated how severe environmental heat can be during major competitions, with exertional heat illness observed despite extensive mitigation efforts (11, 14). Tokyo also demonstrated the operational feasibility of integrating physiological monitoring into event medical operations. However, these systems were not used to guide clinical decision-making, and their implementation should not be interpreted as evidence of a reduction in heat-related illness (11, 15). Subsequent deployments at the Paris 2024 Olympic and Paralympic Games further demonstrated this feasibility (16). A distributed network of meteorological stations were used to measure microclimatic variation directly at the field of play, consistent with known fluctuations within stadium environments that can exceed 10 °C in certain seating zones (17). Athletes voluntarily used validated biometric sensors under medical supervision. Although the data were not used to influence competitive outcomes, the Paris Games confirmed that such systems can operate reliably at scale. This scale aligns with the workforce sizes commonly encountered in high-resource workplaces, where physiological monitoring is more feasible and likely to be adopted.

Across elite-sport research, a consistent lesson is that no single physiological metric such as heart rate, skin temperature, or even core temperature, adequately captures an individual’s heat strain (18, 19). Rather, integration of thermoregulatory, cardiovascular, biomechanical, and environmental data provides a more accurate representation of the athlete–environment interaction (18). Although ingestible thermistors remain the gold standard for core temperature measurement (20), field studies in triathlon (10), ultra-endurance racing (16), and extreme-cold expeditions (21) have shown that multi-sensor systems can demonstrate both the value and limitations of current physiological technologies. Multi-sensor systems provide real-time insights into physiological responses but come with challenges that may impact data quality. Examples include loss of data transmission during swimming events, battery failures in long-duration races, and signal degradation under sweat or personal protective equipment (PPE) (22, 23). These limitations highlight the need for contextual interpretation rather than overreliance on any single metric.

Recognition of these limitations has driven interest in multimodal sensing and algorithmic approaches capable of synthesizing complex physiological datasets. Machine-learning models are increasingly being explored in elite sport to identify patterns of escalating physiological strain using combinations of temperature, cardiovascular, biomechanical, and environmental inputs (24–27). However, these systems remain in the developmental stage. Inter-individual variability, sensor validity concerns, data quality issues, and the limited availability of large outcome datasets continue to constrain the reliability of predictive algorithms (11, 28, 29). For these reasons, elite sport continues to employ human-in-the-loop models, where clinicians interpret physiological data alongside symptoms, environmental conditions, and athletic-specific context rather than deferring to algorithmic outputs alone. This approach aligns with the current governance expectations in sport, which emphasize proportional and ethical use of technology for athlete safety (11, 30, 31).

Crucially, some of the most transferable lessons from elite sport may not be technological but organizational. As physiological monitoring capabilities have expanded, governing bodies and sport organizations have begun to establish frameworks to address data ownership, competitive fairness, privacy, and technological validity (31, 32). This governance framework provides a foundation for occupational implementation, but direct translation is complicated due to substantial differences in athletes and workers.

## Translation: limits, opportunities, and conditions for wearable use in workplaces

### Occupational context differs from sport

While sport science provides valuable insight into thermoregulation and physiological monitoring, direct translation to occupational settings must be approached cautiously because the environments differ fundamentally (33). Workers represent a far more heterogeneous population in terms of age, underlying health status, acclimatization, and physical fitness. They are often exposed to heat for longer durations, experience intermittent or unpredictable work–rest cycles and perform tasks while wearing heavy or impermeable PPE (1, 34–36). Unlike athletes, workers typically do not have continuous medical supervision or immediate access to medical care. These challenges mean that translation from sport must be incremental and embedded within established heat stress prevention practices rather than implemented as a standalone solution. Furthermore, certain worker populations, including migrant workers, undocumented workers, gig economy workers, or workers in South Asia, sub-Saharan Africa, and Latin America, may face heightened vulnerability to extreme heat because they experience not only elevated occupational heat exposure but also social and economic barriers that can limit access to workplace protections, healthcare, and heat mitigation resources (9). These populations may also be at increased risk of workplace exploitation, and these considerations should be incorporated when deploying physiological monitoring systems within a heat illness prevention plan. In addition, obtaining informed consent is critical when physiological monitoring is implemented in occupational settings, as the employer-employee relationship may structurally constrain workers’ ability to freely decline participation, creating the potential for coercive or perceived coercive participation.

### Governance and decision authority

For physiological monitoring to be implemented responsibly, organizations must establish clear governance structures that define data ownership, access, interpretation, and permissible uses (11, 37). Physiological data should never be used for disciplinary action, compensation decisions, employment eligibility determinations, or immigration-related assessments. Instead, monitoring programs should be designed solely to support worker health, safety, and operational decision-making while maintaining appropriate safeguards for worker rights and privacy.

An often overlooked difference between sport and occupational settings is who is responsible for interpreting physiological data and making safety decisions (i.e., analyses and interprets data) (38). In sport, trained physiologists, clinicians, or performance staff—individuals with expertise in thermoregulation and physiology—interpret physiological data and guide return-to-play or workload decisions. In occupational environments, however, decision-making authority may reside with employers, supervisors, safety managers, or industrial hygienists, whose training in thermoregulation varies widely (38). This variability in expertise makes implementing complex physiological monitoring frameworks considerably more challenging. Employers face uncertainty regarding how best to incorporate use of physiological monitoring into routine practice.

### Limitations

Current physiological monitoring approaches in the occupational setting have important limitations. Methods largely rely on single-metric, fixed-threshold principles, whereby workers who reach a predefined physiological limit based on a single metric must stop or modify their work activity (9, 39, 40). These threshold principles are not always linked to individualized risk profiles or task-specific injury likelihood due to the substantial worker heterogeneity in responses to heat (9, 19, 41, 42). Consequently, this approach may generate a substantial number of false positives, where workers are unnecessarily removed from the work environment. Frequent false-positive alerts may undermine confidence in physiological monitoring systems and reduce their perceived value in occupational settings. When removal decisions appear overly conservative or poorly contextualized, employers may perceive physiological monitoring as a productivity constraint rather than a safety enhancement, which limits adoption. Moreover, single-metric, fixed-threshold approaches were often derived from studies of young, physically active men—populations that do not reflect the demographic diversity of today’s global workforce (40, 42–44). Therefore, there is a need to develop individualized thresholds and employ multi-sensor systems that integrate multiple physiological and environmental indicators to provide actionable operational guidance.

### Occupational use cases

Physiological monitoring can add value in specific, well-defined occupational use cases, particularly in the context of heat acclimatization. Heat acclimatization is a physiological adaptation process that occurs during progressive exposure to hot environments and improves tolerance to heat stress (45). In occupational settings, heat acclimatization is commonly implemented using prescriptive ramp-up schedules, in which workers are exposed to a percentage of their usual work duration in the heat and progressively increase to full exposure over several days (40). For example, the NIOSH Criteria for a Recommended Standard: Occupational Exposure to Heat and Hot Environments (2017) document recommends that new and unacclimatized workers should begin at no more than 20% of their usual work shift duration (i.e., 20% rule), whereby heat exposure is increased by no more than 20% each following day.

While prescriptive schedules offer administrative simplicity and clear structure, they rely on several important assumptions, including stable environmental conditions, consistent task demands, homogeneous physiological responses among workers, and exposure occurring under compensable heat stress conditions. In practice, these assumptions are rarely fully met. Environmental heat load can fluctuate substantially across days and shifts, task demands may vary in metabolic intensity, and individual responses to heat are highly heterogeneous (42, 46). As a result, a fixed progression schedule may not adequately reflect true physiological adaptation or strain.

A performance-based heat acclimatization model, supported by physiological monitoring, offers an alternative approach. In this framework, progression is guided by demonstrated heat tolerance rather than predetermined daily exposure increments. Health and safety professionals such as industrial hygienists, supervisors, and physiologist could evaluate pre-shift self-reported readiness alongside on-shift physiological responses to determine whether heat exposure can safely increase or whether additional prevention strategies are warranted. Physiologists monitoring may be especially useful during periods of heightened vigilance, an approach that has been proposed as an alternative to prescriptive heat-acclimatization schedules (47). Under this framework, workers at greater risk of heat strain receive enhanced surveillance and support during heat exposure. This may include new workers, those who are not yet acclimatized, and workers with health conditions or physiological states that may increase susceptibility to heat-related illness, such as diabetes, hypertension, pregnancy, or the postpartum period.

Although heat acclimatization represents a high-yield entry point for physiological monitoring, its utility extends beyond this context. These include characterizing the heat demand associated with specific job tasks, identifying work locations or roles that consistently produce high physiological strain, evaluating the effectiveness of interventions such as cooling technologies or shade structures, and enhancing organizational awareness during extreme heat events. Without physiological monitoring, assessments of intervention effectiveness are often limited to subjective measures such as thermal comfort (37). While thermal comfort is important because it influences behavior, it can also create a false sense of protection. Worker perceptions are shaped by culture, production pressures, and motivation (48, 49). Many workers normalize heat discomfort or suppress behavioral cues to reduce pace or take rest breaks (50, 51). Therefore, physiological monitoring provides a more objective assessment of strain that is less influenced by perception or safety culture (52). Relying solely on subjective assessments of effectiveness may lead to overconfidence in protective strategies and an underestimation of actual heat injury risk. When implemented thoughtfully, wearable monitoring systems can also contribute de-identified, aggregate data to improve organizational understanding of heat exposure patterns over time, thereby supporting continuous improvement in heat risk management practices.

## Physiology-based monitoring adoption framework: applying a tiered strategy

**Figure 1:**
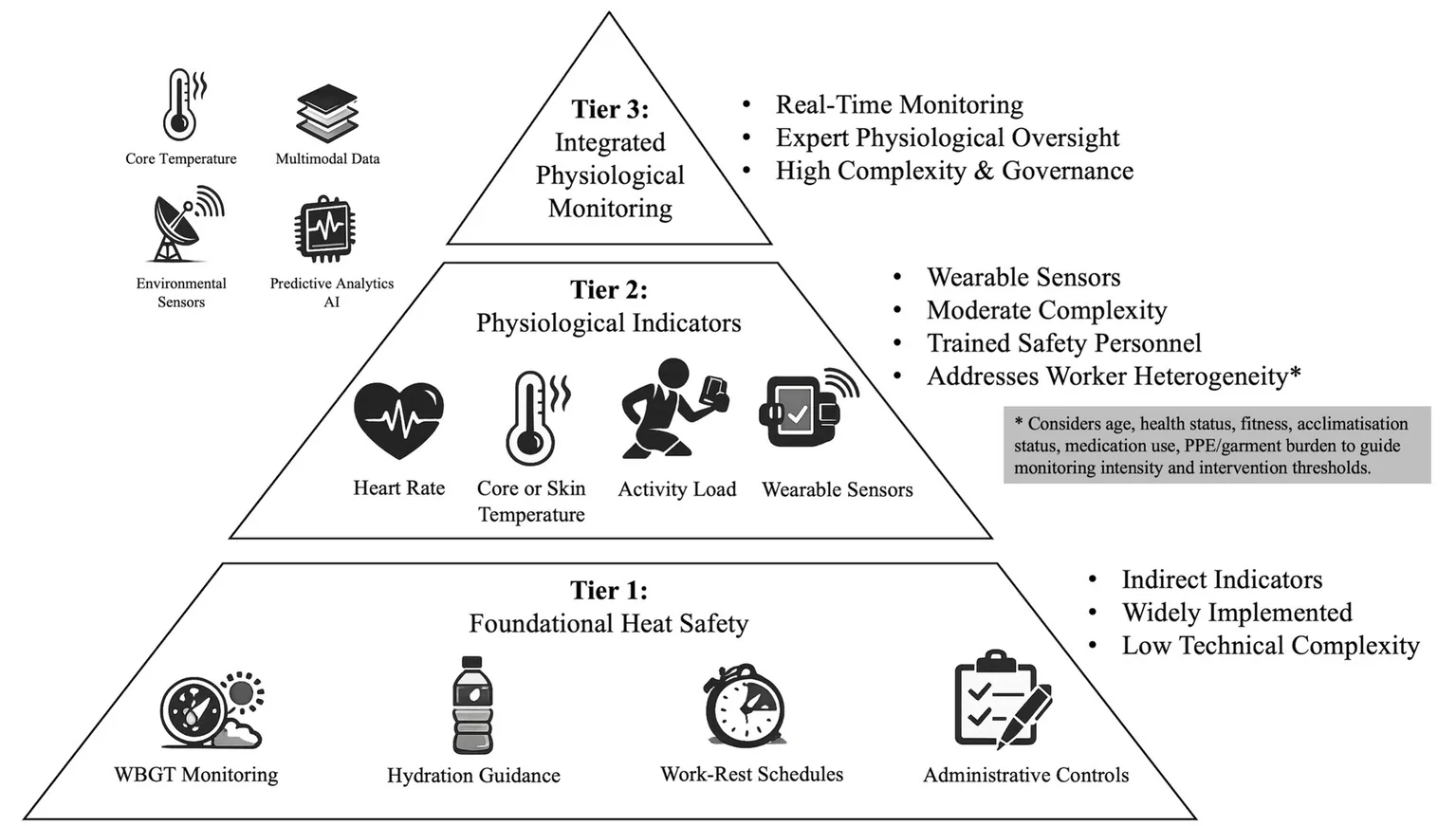
Conceptual framework for translating physiological monitoring from elite sport to occupational heat-safety applications.

Effective implementation of physiological monitoring, particularly complex multi-sensor systems, requires a clearly defined operational framework (see Figure 1). Moreover, the ability to adopt complex multi-sensor systems depends heavily on available resources, personnel, and organizational needs. For example, the use of predictive analytics and multimodal data often requires substantial financial, technological, and personnel resources, thereby limiting its feasibility in middle and low-income occupational settings (9). This presents a particular challenge because workers in low- and middle-income countries experience a disproportionate burden of occupational heat exposure and heat-related illness compared with those in high-income countries, especially in sectors such as agriculture and construction, where physically demanding work is performed under hot environmental conditions (9).

The proposed framework illustrates three levels of adoption of physiological monitoring. While organizations should strive to implement the highest level of physiological monitoring (i.e., Tier 3) whenever feasible, tier selection should be guided by factors such as workforce characteristics, work environment, industry, organizational resources, and readiness to adopt physiological monitoring systems. This framework was developed to illustrate our proposed approach for integrating physiological monitoring into occupational heat illness prevention programs. It provides a foundation for implementation by describing progressively more comprehensive levels of surveillance, with Tier 3 representing the most protective approach based on current evidence. As the evidence base continues to evolve, future research will help refine decision-making frameworks to identify the most appropriate tier for different occupational settings, worker populations, and implementation contexts.

### Tier 1

Tier 1 uses indirect but foundational indicators such as environmental monitoring and administrative controls. These indicators can be integrated with limited resources and do not require expert or trained safety staff.

### Tier 2

Tier 2 integrates wearable sensors to measure physiological indicators that can be monitored and interpreted by trained safety personnel. Wearable physiological monitoring technologies enable the assessment of individual and group responses to a given heat load, helping address one of the primary challenges in occupational heat management: substantial inter-individual variability in physiological responses driven by differences in age, health status, fitness, work intensity, hydration status, and other worker-specific factors. Trained safety staff can utilize wearable sensors to determine when intervention is warranted and allow for ease in assessment during heightened vigilance periods. However, the use of physiological indicators, especially as single metrics require rigorous testing (i.e., validation) to ensure that decisions based on these indicators reflect changes in heat tolerance or increased risk of heat-related injury and illness. This framework should function in parallel with traditional monitoring of heat illness signs and symptoms rather than replacing established safety practices (53). The sensor performance must also be tested in occupational environments, where it may be compromised by sweat, dirt, vibration, PPE, and prolonged shift durations (53, 54). For this reason, it is essential to select devices that have undergone rigorous validation in conditions representative of the intended work setting. Additionally, before implementation, the workplace should be surveyed to determine whether it has adequate connectivity or data infrastructure to support real-time systems.

A major barrier to implementing physiological monitoring is the need to minimize work disruptions. As a result, wearable technologies that estimate physiological parameters are often favored over direct measurements. For example, estimated core temperature derived from wearable sensors is commonly used because direct assessment methods such as ingestible gastrointestinal thermistor pills can be costly, logistically challenging, and difficult to deploy at scale in large work environments (37). Despite these challenges, gold-standard assessments of data should be used when possible.

### Tier 3

Tier 3 represents advanced, real-time multimodal physiological monitoring systems implemented under professional physiological oversight. Similar to Tier 2, these systems require validation prior to deployment; however, Tier 3 also requires specialized expertise and training to interpret physiological data and integrate findings into individualized heat risk management strategies. Workplaces employing workers at elevated risk of heat-related illness, including older adults, pregnant workers, individuals with cardiovascular disease or other relevant health risk factors, workers wearing highly insulating personal protective equipment, and those performing physically demanding work in extreme heat with limited recovery opportunities, may derive the greatest benefit from Tier 3 monitoring. As the evidence base continues to evolve, future research should better define the worker populations and occupational settings for which this level of monitoring provides the greatest value. Moreover, successful adoption of both Tier 2 and Tier 3 depends on establishing appropriate governance structures, including policies addressing data management, privacy, oversight, and the integration of physiological monitoring into occupational heat safety programs.

## Governance: essential conditions for ethical and safe implementation

Governance is central to any attempt to use physiological monitoring in occupational contexts. Globally, occupational heat stress regulatory protection relies only on broad occupational health and safety legislation where employers are required to “provide a safe work environment and mitigate known hazards” (55). Examples of this legislation include OSHA’s general duty clause and EU Directive 89/391/EEC. For guidance on how to control heat exposure, limit heat strain, and use physiological monitoring, employers are directed to non-regulatory guidance documents, recommendations, and consensus standards (i.e., ISO 7243, ISO 7933) (37, 53, 56, 57). While there are several guidance documents related to the use of physiological monitoring to assess heat strain, without strict regulatory boundaries, physiological data could be misused in ways that undermine worker rights or safety. Governance can ensure that physiological data are not used for disciplinary decisions, pay modification, task allocation, or immigration or employment consequences. Data can support productivity management (i.e., identifying workers who may be overworked), but it should not be used to increase productivity at the expense of workers’ health and safety. Workers must have the right to access their own physiological data, and interpretation should be conducted by qualified OEHS or clinical professionals rather than by employers alone. Device validation must follow established standards for accuracy, reliability, and “fit-for-purpose” performance. Equity considerations are essential: age, comorbidity burden, sex differences, and underlying health status influence heat responses (42), and systems must not disadvantage already vulnerable worker groups. If governance is weak, monitoring risks may outweigh the benefits—a concern repeatedly highlighted in both occupational and sports technology ethics literature (7, 11). The proposed adoption framework serves as a method to address the shortcomings and limited governance set in place.

## Future directions

The widespread adoption of Tier 2 and Tier 3 approaches will require additional research across several key areas. First, wearable sensors must be validated under conditions that reflect the realities of physically demanding, intermittent work in hot environments and within populations representative of the workforce. Second, physiological thresholds should move beyond single-metric approaches and be linked to clear, actionable operational guidance that enables decision-makers to determine when work modifications, additional rest, or other interventions are warranted. Further research is also needed to develop and validate machine-learning algorithms that integrate multiple physiological and environmental inputs, while establishing appropriate governance frameworks to address data management, privacy, and decision-making. Addressing these research priorities will facilitate broader adoption of physiological monitoring systems while ensuring appropriate safeguards are in place to protect workers. Moreover, research must be performed in high-priority populations at greatest risk, including workers in construction, agriculture, and migrant worker populations, to ensure these technologies are equitable, effective, and applicable where they are needed most (9).

## Conclusion

Exertional heat stress will continue to pose significant challenges as global temperatures rise. Elite sport has served as an important testing ground for advanced monitoring technologies, demonstrating feasibility but also revealing essential limitations. Translation to occupational settings is feasible, but only when guided by an adoption framework, strong governance, realistic expectations, and rigorous validation. Wearable physiological monitoring should function as an adjunct to established heat-safety practices rather than a replacement for environmental monitoring, administrative controls, or clinical judgment.

## Data Availability

The original contributions presented in the study are included in the article, further inquiries can be directed to the corresponding author.
